# Draft Genome Sequence of the Psychrophilic and Alkaliphilic *Rhodonellum psychrophilum* Strain GCM71^T^

**DOI:** 10.1128/genomeA.01014-13

**Published:** 2013-12-05

**Authors:** Aviaja L. Hauptmann, Mikkel A. Glaring, Peter F. Hallin, Anders Priemé, Peter Stougaard

**Affiliations:** Department of Plant and Environmental Sciences, University of Copenhagen, Copenhagen, Denmarka; Novozymes A/S, Bagsvaerd, Denmarkb; Department of Biology, University of Copenhagen, Copenhagen, Denmarkc

## Abstract

*Rhodonellum psychrophilum* GCM71^T^, isolated from the cold and alkaline submarine ikaite columns in the Ikka Fjord in Greenland, displays optimal growth at 5 to 10°C and pH 10. Here, we report the draft genome sequence of this strain, which may provide insight into the mechanisms of adaptation to these extreme conditions.

## GENOME ANNOUNCEMENT

Cold and alkaline environments are rare on Earth, and knowledge regarding the organisms that thrive in such polyextreme environments is very limited. The submarine ikaite tufa columns in the Ikka Fjord in southwest Greenland (61°11′N, 48°01′W) represent a unique, permanently cold (4 to 6°C), and alkaline (>pH 10) environment (1), and they have been shown to harbor a diverse bacterial community adapted to these conditions (2–4). A large number of bacteria from the ikaite columns have previously been isolated, and the majority of these are psychrotolerant and alkaliphilic or alkalitolerant (2–4). Although a significant number of the isolates display alkaliphilic and psychrotolerant growth properties, only very few true alkaliphilic psychrophiles have been identified. One isolate, *Rhodonellum psychrophilum* GCM71^T^, was shown to be truly alkaliphilic and psychrophilic, displaying optimal growth at temperatures between 5°C and 10°C and at pH 9.2 to 10 (5). Thus, *R. psychrophilum* is one of very few polyextremophilic bacteria adapted to low-temperature and high-pH conditions, and in this report, we describe the annotated draft genome sequence of *R. psychrophilum* GCM71^T^, the first genome sequence of an organism adapted to these conditions. The genome sequence is part of research looking into polyextremophily and genome mining for enzymes that are adapted to alkaline and low-temperature conditions.

Genomic DNA was isolated from a liquid culture of *R. psychrophilum* GCM71^T^ growing in R2 broth medium (3). The genome sequence was obtained by Illumina HiSeq 2000 paired-end sequencing. Assembly was performed using IDBA version 0.19-paired (6), resulting in 199 contigs with a mean length of 25,814 bp and an N_50_ contig length of 69,824 bp. The final average coverage was 485-fold. The draft genome sequence presented here is 5,137,147 bp, with a G+C content of 41.8%. Annotation of the genome through the Rapid Annotations using Subsystems Technology (RAST) server (7) resulted in 5,111 predicted coding sequences and 33 RNA sequences, of which one is the complete 16S rRNA gene sequence. The closest relative with a validly published name for which a genome sequence exists is *Belliella baltica* type strain BA134 (DSM 15883), isolated from the surface waters of the Baltic Sea (8). An independent draft genome sequence of *R. psychrophilum* has recently been released as part of the Genomic Encyclopedia of Type Strains project at the DOE Joint Genome Institute (GenBank accession no. ARMB00000000).

### Nucleotide sequence accession numbers.

This whole-genome shotgun project has been deposited at DDBJ/EMBL/GenBank under the accession no. AWXR00000000. The version described in this paper is the first version, AWXR01000000.
